# Removal of Ampicillin with Nitrifying Cultures in a SBR Reactor

**DOI:** 10.1007/s12010-024-05165-1

**Published:** 2025-01-09

**Authors:** Daniel Maturano-Carrera, Omar Oltehua-López, Flor de María Cuervo-López, Anne-Claire Texier

**Affiliations:** Department of Biotechnology-CBS, Metropolitan Autonomous University Iztapalapa, Av. Ferrocarril San Rafael Atlixco 186, 09310 Mexico City, Mexico

**Keywords:** Ampicillin, Biosorption, Cometabolic biodegradation, Nitrifying sludge, Sequencing batch reactor

## Abstract

The presence of antibiotics in wastewater discharges significantly affects the environment, mainly due to the generation of bacterial populations with multiple antibiotic resistances. The cometabolic capacity of nitrifying sludge to simultaneously remove ammonium (NH_4_^+^) and emerging organic contaminants (EOCs), including antibiotics, has been reported. In the present study, the removal capacity of 50 mg ampicillin (AMP)/L by nitrifying cultures associated with biosorption and biotransformation processes was evaluated in a sequencing batch reactor (SBR) system. The contribution of nitrifying enzymes (ammonium monooxygenase (AMO) and nitrite oxidoreductase (NOR)) and β-lactamases in AMP biodegradation was evaluated using specific inhibitors in batch cultures. AMP was 100% eliminated after 5 h since the first cycle of operation. The sludge maintained its ammonium oxidizing capacity with the total consumption of 102.0 ± 2.5 mg NH_4_^+^-N/L in 9 h, however, the addition of AMP altered the nitrite-oxidizing process of nitrification, recovering 30 cycles later at both physiological and kinetic level. The kinetic activity of the nitrifying sludge improved along the operating cycles for both AMP removal and nitrification processes. The elimination of 24% AMP was attributed to the biosorption process and 76% to biotransformation, wherein the AMO enzyme contributed 95% to its biodegradation. Finally, the repeated exposure of the sludge to AMP for 72 operating cycles (36 days) was not sufficient to detect β-lactamase activity. The cometabolic ability of ammonium-oxidizing bacteria for biodegrading AMP could be employed for bioremediation of wastewater.

**Graphical Abstract:**

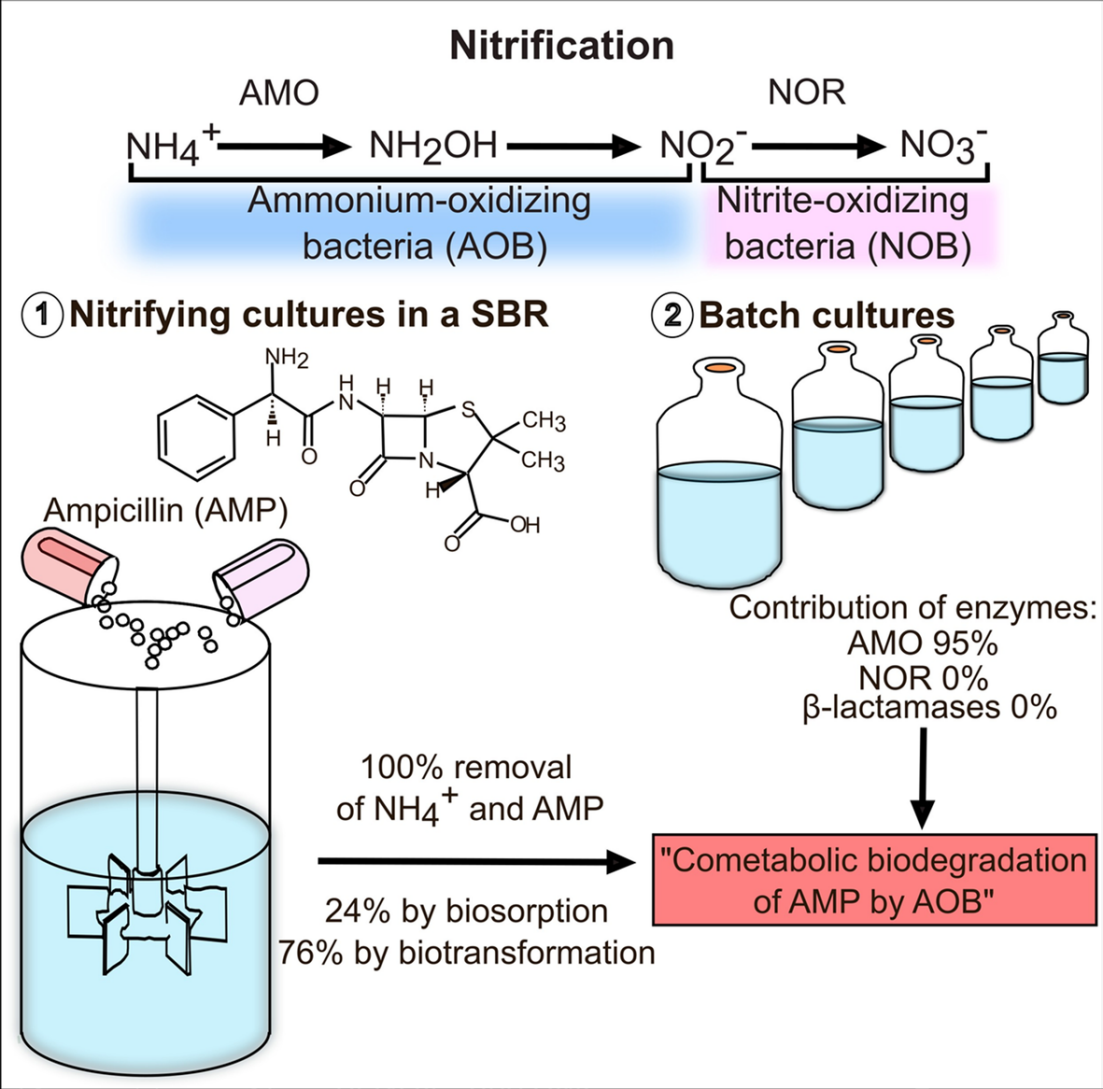

## Introduction

Since the discovery of penicillin, the use of antibiotics as a treatment for various bacterial diseases worldwide has been constantly increasing. The production of antibiotics around the world exceeds values of 100,000 tons per year, including groups of sulfonamides, tetracyclines, fluoroquinones, macrolides, and β-lactams [1]. Besides, it is estimated that antibiotics for veterinary use will have an increase in their production of 30% by the year 2030 [2]. Chemically, antibiotics have complex structures which make their elimination difficult, being the main problem their low metabolism by the human and animal body, where around 30% and up to 90% of antibiotics are not modified after their ingestion and discarded in wastewater discharges [3, 4]. Today, the presence of antibiotics in wastewater, surface water, and even oceans has been confirmed, at concentrations ranging from ng/L to mg/L in surface water and pharmaceutical discharges, respectively [5, 6]. Ampicillin (AMP) is one of the most widely used antibiotics, belonging to the group of β-lactams, which has been inadequately consumed and has become an environmental problem worldwide [7]. Likewise, recent studies have shown that antibiotics can alter biological processes during wastewater treatment [8–10] and generate bacterial resistance [11], producing antibiotic-inhibiting enzymes such as β-lactamases [12, 13].

In recent years, the nitrification biological process has been widely studied for the simultaneous removal of ammonium and emerging organic pollutants and drugs from wastewater [14–16]. However, the interactions between nitrifying microorganisms and antibiotics are not yet well-described. The role of nitrifying enzymes and bacterial resistance enzymes during antibiotics degradation in biological processes needs to be more investigated [17–19]. In the two-step nitrifying process, ammonium-oxidizing bacteria (AOB) oxidize ammonium to nitrite and nitrite-oxidizing bacteria (NOB) convert the formed nitrite to nitrate. In its first stage, the ammonium oxidation is catalyzed by the enzyme ammonium monooxygenase (AMO), while in its second stage, the nitrite oxidation is carried out by the enzyme nitrite oxide reductase (NOR). Currently, it has been shown that the elimination of some antibiotics is associated with the AMO enzyme activity [16, 17]. Wang et al. [20] reported high antibiotic removal efficiencies by nitrifying consortia with 94.6 and 75.4% biodegradation of cephalexin and sulfadiazine, respectively associated with cometabolic degradation by AOB and non-specific activity of AMO enzyme. Very few reports assessed the role of NOB and NOR enzyme in the elimination of aromatic compounds and antibiotics [21]. In activated sludge processes, the biodegradation of β-lactam antibiotics can be initiated by β-lactamases, catalyzing the hydrolysis of the β-lactam ring [6]. Then, it is also needed to evaluate in nitrifying reactors the contribution of enzymes involved in antibiotic resistance such as the activity of β-lactamase enzymes [18]. Magee et al. [22] detected the presence of β-lactams such as amoxicillin and AMP in sewage sludge from wastewater treatment plants after storage at low concentrations of 1.0 and 14.8 ng/g dry weight, respectively. However, results of studies on β-lactams biosorption in activated sludge processes can vary greatly and more research in this sense is needed [6]. During the exposure of sludge to antibiotics, the first mechanism of antibiotics elimination is adsorption, followed by possible biotransformation. In fact, biosorption has been reported as one of the main processes to remove antibiotics in biological wastewater treatment [6, 17]. In the activated sludge process, Wang et al. [23] reported the elimination of antibiotics from the fluoroquinolone group in a range of 78–91% by biosorption and 9–22% by biotransformation.

Although AOB are able to eliminate antibiotics, these bacteria can be sensitive to the toxic and inhibitory effects of these compounds, and consequently, nitrification performance can be altered in activated sludge [9]. Guo et al. [24] reported lower ammonium removal efficiencies when adding 10mg sulfamethoxazole/L to a sludge obtained from a wastewater treatment plant in a SBR. On the other hand, Katipoglu-Yazan et al. [25] indicated that the use of 200mg tetracycline/L stopped the nitrite oxidative process in a mixed heterotrophic and autotrophic culture. Therefore, the presence of antibiotics in nitrifying cultures can alter the nitrification process to a different degree according to their concentration, observing accumulation of ammonium or nitrite depending on the affected oxidative process. With respect to ampicillin, Lopez et al. [26] assessed its short-term toxic effect on enriched nitrifying cultures, reporting an EC_50_ of 23.7 mg/L. Yu et al. [27] observed the inhibitory effects of AMP (10–30 mg/L) on the nitrifying activity of an activated sludge in a SBR, determining a significant decrease in the specific ammonium- and nitrite-oxidizing rates. The authors related these effects to an inhibition on AMO and NOR enzymatic activities and a decrease of the relative abundance of *Nitrosomonas* and *Nitrospira*. Recently, Esquivel-Mackenzie et al. [28] evidenced that the repetitive exposure of a stabilized nitrifying sludge to 14.4 mg AMP/L in an SBR system caused an inhibition of up to 67% on the nitrite-oxidizing process. However, the NOB showed a physiological adaptation for recovering a complete nitrite-oxidizing process along the operation cycles of the reactor, associated with an increase in *Nitrospira* abundance. As previously described, there are many studies on the effects of AMP on the nitrifying processes; however, very few information is still available on the processes involved in its removal. It has been previously reported that AMP can be removed in nitrifying cultures [28], but more investigation is required to understand better how the processes of biosorption and biotransformation contribute to AMP removal in biological reactors. The enzymes involved in the AMP removal in nitrifying bioreactors should be studied.

The main objective was to evaluate the AMP removal and nitrification processes throughout the operating cycles of a SBR reactor, as well as the contribution of AMO, NOR, and β-lactamase enzymes in AMP biodegradation using specific enzymatic inhibitors in batch assays.

## Materials and Methods

### Operation of the Nitrifying SBR Reactor

The sludge was collected from manure before the acclimatization and stabilization in a laboratory reactor fed with a synthetic nitrogen-rich medium for 30 years. The nitrifying reactor was operated continuously in steady state and fed with a N medium composed of (g/L): (NH_4_)_2_SO_4_ 1.73, NH_4_Cl 1.39, KH_2_PO_4_ 2.73, MgSO_4_ 1.26, and NaCl 1.0 and a C medium composed of (g/L): NaHCO_3_ 12.83 and CaCl_2_ 0.005 [15]. Bicarbonate was used as a carbon source and pH buffer. The SBR reactor (1.6 L) was inoculated with an initial concentration of 320 ± 10 mg of microbial protein/L, which was kept constant during all operation cycles. The experimentation in the SBR reactor was divided into two phases: (1) nitrifying control without AMP (operation cycles 1–15) and (2) nitrifying cultures with 50mg AMP/L (cycles 16–87). During this last phase, the sludge was extracted during cycles 17, 52, and 87 for being used as inoculum in batch culture experimentation. In the SBR reactor, each operation cycle had a total duration of 12 h: feeding (5 min), reaction (11 h), sedimentation (45 min), and drainage (10 min). The following operating conditions were maintained: initial ammonium concentration of 100.2 ± 2.5 mg NH_4_^+^-N/L, the composition of the N and C media was the same as that used in the continuous reactor, adjusting the C/N ratio to 2.5, stirring at 200 rpm, constant aeration, average dissolved oxygen (DO) concentration of 3.5 ± 0.5 mg/L, temperature of 30 ± 2 °C, pH of 7.6 ± 0.3. Samples were taken at different times during the reaction phase of various operation cycles, centrifuged at 4000 rpm for 2 min, and filtered (0.2 μm) before analysis. For each evaluation period (without AMP: cycles 1–15; with AMP addition: cycles 16–45, cycles 46–75, and cycles 76–87), the reported values represent the mean ± standard deviation from at least three different kinetic profiles. During the evaluation period of cycles 1–15, three nitrifying kinetic profiles were performed at cycle 15. For the evaluation periods with AMP at cycles 16–45, cycles 46–75, and cycles 76–87, three different kinetic assessments were realized at each of the following cycles: 16, 26, 36, 46, 56, 66, 76, and 86.

### Batch Culture Experimentation

Serological bottles with a nominal volume of 160 mL and an operating volume of 100 mL were used for performing batch cultures. The synthetic N and C media were the same as those used in the SBR reactor, adjusting their composition for obtaining an initial ammonium concentration of 49.2 ± 2.5 mg NH_4_^+^-N/L and a C/N relation of 2.5. Bottles were inoculated with the 120 ± 5mg microbial protein/L of the sludge collected from the SBR reactor at cycles 17, 52, or 87. The experimentation in batch cultures was divided into four parts: (1) abiotic assays without microbial biomass; (2) AMP biosorption assays where the aerobic biological activity of the nitrifying sludge was stopped by displacing the oxygen present in the headspace and liquid medium of the serological bottles with a continuous stream of N_2_ for 5 min according to the methodology proposed by Hamon et al. [29] and Ramírez Muñoz et al. [30]; (3) control cultures with active biomass, oxygen, and AMP (25 mg/L) (biosorption and biotransformation processes); and (4) contribution assays of nitrifying enzymes and bacterial resistance activity by using enzymatic inhibitors: control without inhibitors, with 50mg allylthiourea (ATU)/L as specific inhibitor of AMO, with 80mg sodium chlorate (NaClO_3_)/L as specific inhibitor of NOR, or with 150mg sulbactam/L as specific inhibitor of β-lactamases. The experimental conditions were maintained as follows: C/N relation of 2.5, stirring at 200 rpm, temperature of 30 ± 2 °C, and average pH of 7.4 ± 0.3. The initial DO concentration was of 3.5 ± 0.5 mg/L by saturating with oxygen the headspace and liquid phases of the bottles. For that, a current of oxygen gas (99.6% of purity) was injected in the medium and the headspace for 3 min. The bottles were sealed with rubber stoppers and aluminum crimp caps. Under each experimental condition, the 10h batch assays were performed by triplicate. Three independent samples were taken every hour, centrifuged at 4000 rpm for 2 min, and filtered (0.2 μm) before analysis.

### Analytical Methods

For the analysis of AMP, a liquid chromatograph was used (Perkin Elmer Series 200, USA) at a wavelength of 230 nm with a C18 column (stationary phase Microsorb 100, 4.6 × 150 mm, Varian, USA) and a flow of mobile phase of 1 mL/min [30]. Selective electrodes were employed for the analysis of ammonium (Phoenix Electrode Company, USA), DO (DO HI 98186 Hanna, Romania), and pH (Digi-Sense digital pH, USA) [31]. For the quantification of nitrite and nitrate, a liquid chromatograph was used (Perkin Elmer Series 200, USA) at a wavelength of 214 nm with an ion exchange column (IC-Pak Anion HC, 4.6 × 150 mm, Waters, USA) and a flow of mobile phase of 2 mL/min [15]. The microbial biomass concentration was measured through protein determination. The modified Lowry colorimetric method was used to determine the microbial protein concentration in the reactor and the serological bottles [32, 33]. In all cases, standard curves were made in triplicate and analyzed as linear regressions, obtaining coefficients of variation for slopes lower than 5% and coefficients of determination (*r*^2^) higher than 0.99.

### Evaluation of the Physiological and Kinetic Activity of the Nitrifying Cultures

The nitrifying sludge activity was physiologically characterized in terms of AMP and NH_4_^+^ consumption efficiencies (E_AMP_, [mg AMP removed/mg AMP added] × 100 and ENH_4_^+^, [mg NH_4_^+^-N consumed/mg NH_4_^+^-N fed] × 100) and product formation yields (YNO_2_^−^, [mg NO_2_^−^-N produced/mg NH_4_^+^-N consumed] and YNO_3_^−^, [mg NO_3_^−^-N produced/mg NH_4_^+^-N consumed]). The kinetic behavior was assessed using the lag phases (λ), the specific uptake rates of AMP (q_AMP_, [mg AMP/mg microbial protein h]) and NH_4_^+^ (qNH_4_^+^, [mg NH_4_^+^-N/mg microbial protein h]), and the specific formation rates of NO_2_^−^ (qNO_2_^−^, [mg NO_2_^−^-N/mg microbial protein h]) and NO_3_^−^ (qNO_3_^−^, [mg NO_3_^−^-N/mg microbial protein h]). *λ* values and specific rates were determined by using the adjusted Gompertz model and the package NCSS 2021 software as previously described by Maturano‑Carrera et al. [31]. Concentrations of AMP and NH_4_^+^ consumed, NO_2_^−^ and NO_3_^−^ formed *Y*(*t*) (mg/L), at time *t* (h), can be expressed as a function of time according to Eq. (1) where the parameters *A*, *B*, and *C* represent the maximum concentration of substrate consumed or product formed (mg/L), the volumetric rate of substrate consumed or product formed (mg/L h), and the inflection point of the curve (h), respectively. Lag phases (*λ*, *h*), maximum volumetric consumption or formation rates (Vmax, mg/L h), and specific rates (*q*, mg/mg microbial protein h) were determined as previously described by Maturano‑Carrera et al. [31]. The *r*^2^ values (*r*^2^ > 0.98) and the model significance were used as adjusting criterion.

1 $$Y\left(t\right)=A\;exp\;\left(-exp\;\left(-B\;\left(\left(t\right)-C\right)\right)\right)$$

## Results and Discussion

### Ampicillin Removal in the SBR Reactor

**Fig. 1:**
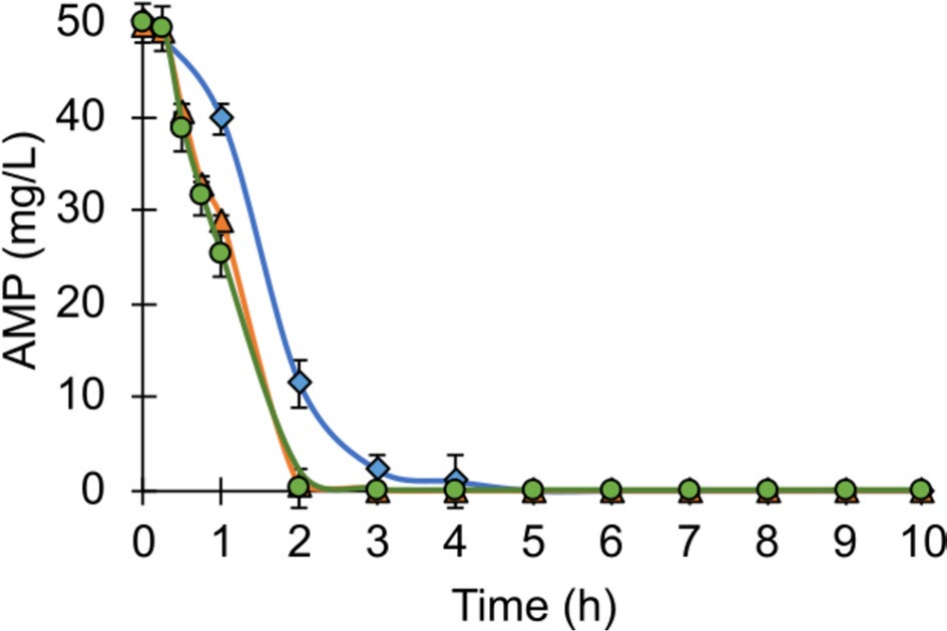
Kinetic profiles of AMP removal in the SBR reactor. Cycles 16–45 (blue diamond), cycles 46–75 (orange triangle), and cycles 76–87 (green circle)

The AMP removal profiles obtained in the SBR reactor are shown in Fig. 1. Since the first addition of the antibiotic (cycle 16), the nitrifying sludge was able to totally remove 50mg AMP/L. During cycles 16–45, the time required for the total elimination of ampicillin was 5 h, while in cycles 46–75 and cycles 76–87, only 2 h was needed (Table 1). The decrease in the time for the complete elimination of AMP along the operation cycles was related to a decrease of the average lag phases for initiating its consumption and an increase of the specific rates of AMP removal (Table 2). The *λ* values decreased 60% in the last evaluation period during cycles 76–87 compared to the first evaluation period of the antibiotic addition (cycles 16–45) while *q*_AMP_ values increased 10% between the same periods (Table 2). The results obtained in this study coincide with those reported by Esquivel-Mackenzie et al. [28] where 14.4 mg AMP/L was completely consumed by a nitrifying consortium in a time of 0.5 h. The difference between the times required to consume AMP could be due to the fact that in the present work, the initial AMP concentration was 3.5 times higher than that used by Esquivel-Mackenzie et al. [28]. In aerobic granules, Wang et al. [34] reported a 97% removal of 10 mg AMP/L in 6 h. The higher capacities of nitrifying sludge to remove AMP might be related to the role of ammonium-oxidizing microorganisms in the biodegradation process, as they have been previously shown to enhance the removal of trace organic chemicals and pharmaceuticals [14, 16]. Besides, the improvement in the AMP removal observed throughout the cycles in the present study could be related to a higher metabolic capacity for biodegrading the antibiotic. This has been previously reported by various authors for other organic molecules in nitrifying systems [35] where changes in the microbial community [36] and the enzymatic activity are involved [37]. However, information on the processes and enzymes involved in the removal of antibiotics in nitrifying bioreactors is still very limited [17].

**Table 1:** Physiological activity of the nitrifying sludge

Stage	Cycles	Time required for total AMP removal (h)	E_AMP_ (%)	Time required for total NH_4_^+^ consumption (h)	ENH_4_^+^ (%)	YNO_2_^−^	YNO_3_^−^
Without AMP	1–15	-	-	9	99.9 ± 0.1	0	0.99 ± 0.01
With AMP addition	16–45	5	99.9 ± 0.1	9	99.8 ± 0.1	0.24 ± 0.01	0.75 ± 0.02
	46–75	2	99.9 ± 0.1	6	99.9 ± 0.1	0	0.99 ± 0.01
	76–87	2	99.9 ± 0.1	5	99.9 ± 0.1	0	0.99 ± 0.01

*E*_*AMP*_ ampicillin removal efficiency (mg AMP removed/mg AMP fed) × 100, *ENH*_*4*_^+^ ammonium consumption efficiency (mg NH_4_^+^-N consumed/mg NH_4_^+^-N fed) × 100, *YNO*_*2*_^*−*^ nitrite production yield (mg NO_2_^−^-N/mg NH_4_^+^-N consumed), *YNO*_*3*_^*−*^ nitrate production yield (mg NO_3_^−^-N/mg NH_4_^+^-N consumed)

**Table 2:** Kinetic activity of the nitrifying sludge

Stage	Cycles	AMP removal		NH_4_^+^ consumption		NO_3_^−^ formation	
		λ	q_AMP_	λ	qNH_4_^+^	λ	qNO_3_^−^
Without AMP	1–15	-	-	2.9 ± 0.1	0.102 ± 0.002	3.1 ± 0.1	0.125 ± 0.001
With AMP addition	16–45	0.05 ± 0.01	0.220 ± 0.002	2.9 ± 0.1	0.101 ± 0.002	2.9 ± 0.1	0.084 ± 0.001
	46–75	0.03 ± 0.01	0.233 ± 0.001	2.0 ± 0.2	0.109 ± 0.002	2.4 ± 0.1	0.124 ± 0.002
	76–87	0.02 ± 0.01	0.242 ± 0.002	0.4 ± 0.2	0.111 ± 0.002	1.2 ± 0.1	0.131 ± 0.001

*λ* lag phase (h), *q*_*AMP*_ specific removal rate of AMP (mg AMP/mg microbial protein h), *qNH*_*4*_^+^ specific consumption rate of ammonium (mg NH_4_^+^-N consumed/mg microbial protein h), *qNO*_*3*_^*−*^ specific production rate of nitrate (mg NO_3_^−^-N formed/mg microbial protein h)

### Ampicillin Removal by Biosorption and Biotransformation Processes

**Fig. 2:**
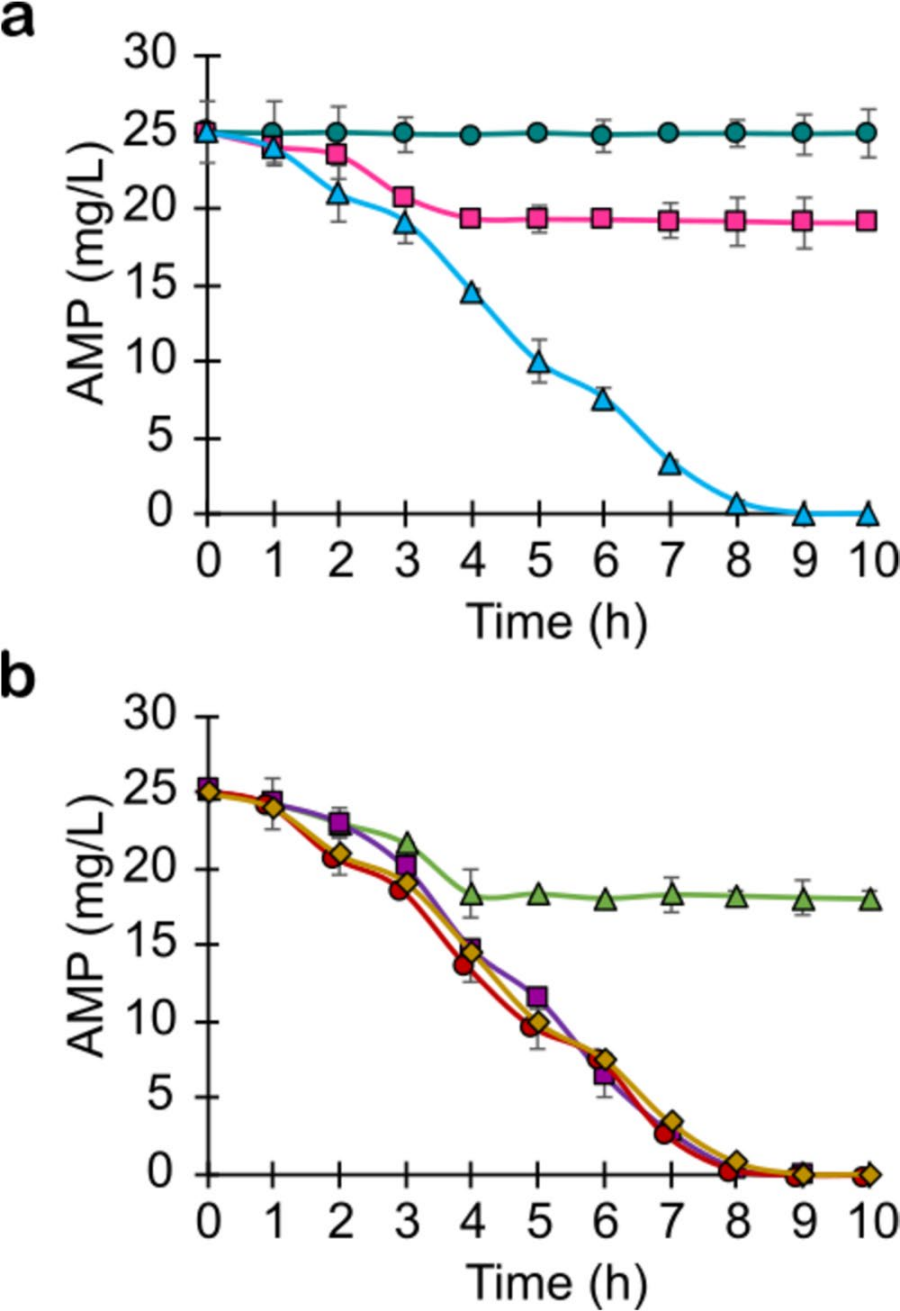
Kinetic profiles of AMP removal in serological bottles (average data obtained in triplicate batch experiments with biomass collected from the SBR at cycles 17, 52, or 87). **a** Abiotic process (green circle), biosorption process (pink square), biosorption and biotransformation processes (blue triangle). **b** Contribution assays of nitrifying enzymes and bacterial resistance with enzymatic inhibitors: control cultures without inhibitors (yellow diamond), allylthiourea (AMO) (green triangle), sodium chlorate (NOR) (violet square), sulbactam (β-lactamases) (red circle)

The abiotic, biosorption, and biotransformation processes potentially responsible for AMP removal in the nitrifying cultures were evaluated (Fig. 2a). It is important to emphasize that results presented in Fig. 2a correspond to the average data obtained in the different batch experiments performed with biomass collected from the SBR at the following operating cycles: 17, 52, or 87. In all cases, the kinetic profiles of AMP removal were similar, showing that the contribution of biosorption and biotransformation processes in the antibiotic elimination did not change along the cycles. Results from batch assays without addition of biomass (abiotic process) and with 25 mg AMP/L demonstrated that under these experimental conditions, the antibiotic was not eliminated by physicochemical processes during the 10 h of evaluation. On the other hand, results from batch experiments with biomass and saturation of the headspace and liquid medium with N_2_ (biosorption process) indicated that 24% of AMP was removed by biosorption. Batch cultures with active biomass and oxygen (biosorption and biotransformation processes) demonstrated a consumption of 100% of the AMP, indicating that 76% of the antibiotic removal was associated with biotransformation. In the present study, biotransformation was the main process for AMP removal by nitrifying sludge, in contrast with the results of Li and Zhang [38] where the major removal route for AMP was adsorption rather than biodegradation in an activated sludge process. In addition to the effect of the antibiotics and their physicochemical properties, the adsorption of antibiotics onto biological sludge depends on the operating conditions of the biological treatment systems and the physicochemical properties of the sludge as its metabolic capacity for biodegrading them. Consequently, results from studies on the biosorption of penicillin antibiotics in biological wastewater treatment systems varied greatly [6].

### Participating Enzymes in AMP Consumption

Within the elimination of AMP by biotransformation, the contribution of the nitrifying enzymes (AMO, NOR) and β-lactamases activity was assessed (Fig. 2b). Comparing with the kinetic profile of AMP removal in control cultures without inhibitors, the AMP consumption profile in assays with ATU as specific inhibitor of the AMO enzyme showed a lower consumption of the antibiotic. Only 28% of AMP was removed from the 10h cultures performed with ATU. This would correspond to 24% of the fraction eliminated by biosorption (Fig. 2a) and 4% by biological activity (by heterotrophic bacteria as AOB were inhibited). These results showed that the AMO enzyme contributed 72% to AMP removal, representing 95% of the AMP biodegraded fraction. On the other hand, the evaluation of the AMP removal with sodium chlorate as inhibitor of the NOR enzyme demonstrated a total consumption of the antibiotic, similarly to that obtained in control cultures without inhibitors. Then, there was no contribution of the NOR enzymatic activity in AMP consumption. Finally, cultures with sulbactam as inhibitor of β-lactamases also showed a similar AMP consumption profile as obtained in control cultures without inhibitors, indicating that β-lactamase activity was not involved in AMP biotransformation under the experimental conditions used. As the profiles presented in Fig. 2b are the average data from three different experiments realized with biomass proceeding from the SBR at cycles 17, 52, or 87, respectively, it can be concluded that no bacterial resistance by β-lactamases activity was detected even after 72 cycles of repeated exposure of the sludge to AMP. This might be partly due to the high contribution of AMO in AMP biotransformation obtained under the established nitrifying conditions. Biodegradation mechanisms and pathways of antibiotics in biological wastewater treatment systems are currently under study [6, 19]. There are still few studies on biodegradation mechanisms of β-lactams in biological processes, even less under nitrifying conditions. The AMO has been reported as involved in the biodegradation of various pharmaceuticals and antibiotics, catalyzing hydroxylation as the main reaction [16, 17]. In the present study, it is demonstrated that 95% of the AMP biodegradation in nitrifying cultures depended on the activity of the AMO, which would initiate the biotransformation process. It has been shown that the participation of AMO in the cometabolic biodegradation of organic contaminants varied according to the tested molecule, the nitrifying activity of the sludge used, and the experimental conditions, including the availability of ammonium as growth-substrate and energy source for AOB [15, 39, 40].

### Nitrification Performance of the SBR Reactor Fed with Ampicillin

**Fig. 3:**
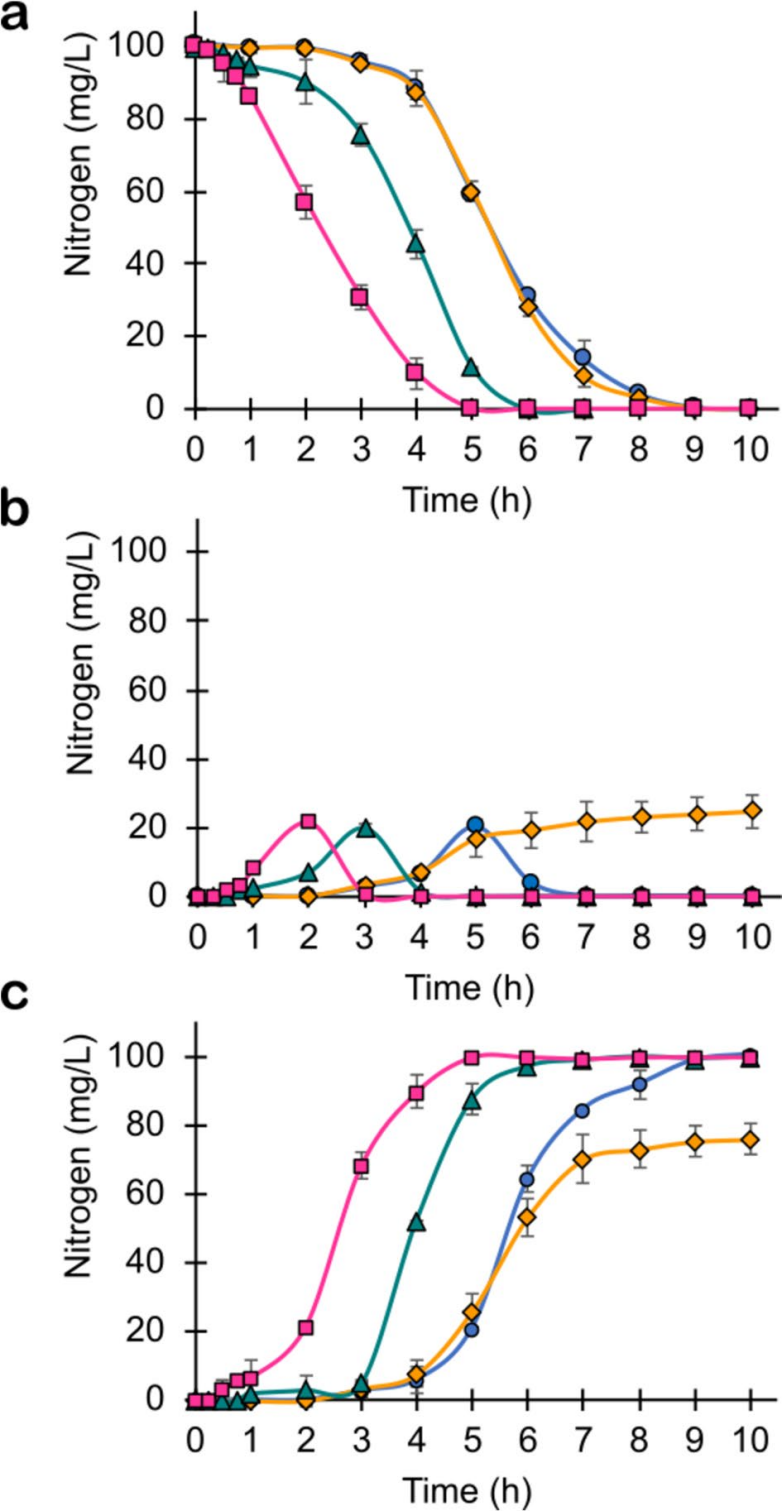
Nitrifying performance of the sludge during the AMP addition in the SBR reactor: **a** ammonium consumption profiles; **b** nitrite formation profiles; **c** nitrate formation profiles. Cycles 1–15 (control without AMP addition) (blue circle), cycles 16–45 (orange diamond), cycles 46–75 (green triangle), and cycles 76–87 (pink square)

Kinetic profiles of nitrifying activity during the first 15 operation cycles without addition of AMP are shown in Fig. 3. Since the first operation cycle in the SBR reactor and up to cycle 15, high ENH_4_^+^ values of 99.9 ± 0.1% were obtained, requiring a time of 9 h for total ammonium consumption (Fig. 3a; Table 1). NO_2_^−^-N transiently accumulated, reaching a maximum concentration of 21.1 mg N/L at 5 h and a YNO_2_^−^ of zero from 7 h of culture (Fig. 3b; Table 1). While this first metabolite was produced transiently, there was an oxidation to NO_3_^−^-N, obtaining a high final YNO_3_^−^ of 0.99 ± 0.01 mg NO_3_^−^-N/mg NH_4_^+^-N consumed (Fig. 3c; Table 1). Physiologically, the nitrifying process was complete. Kinetic behavior of the sludge was characterized through lag phases and specific rates for NH_4_^+^ consumption and NO_3_^−^ formation for taking them as control values prior to the addition of AMP (Table 2). Under these parameters, it was confirmed that the nitrifying process within the SBR reactor remained in a steady state.

After 15 cycles of operation of the SBR reactor, the addition of 50 mg AMP/L was started from cycle 16 to cycle 87 (Fig. 3). Ammonium consumption profiles did not change with antibiotic addition during cycles 16 to 45 (Fig. 3a). ENH_4_^+^ remained high at 99.8 ± 0.1% (Table 1) while the lag phases and specific rates of NH_4_^+^ consumption values continued like those obtained during the control period without antibiotic (Table 2). Therefore, the presence of AMP did not physiologically and kinetically affect the ammonium oxidative process. During evaluation periods of cycles 46–75 and cycles 76–87, ammonium consumption improved, and the time required for total NH_4_^+^ consumption dropped to 6 and 5 h, respectively (Fig. 3a; Table 1). As shown in Table 2, with respect to the control value, the qNH_4_^+^ increased up to 8.8% whereas λ decreased up to 86% at cycles 76–87. These results showed that in addition to the fact that AOB were not sensitive to AMP under the experimental conditions used, their ammonium oxidizing capacity improved throughout the operation cycles. This may be associated with the improving AMP removal along the SBR cycles through its cometabolic biotransformation by AMO. Both ammonium consumption and AMP removal processes initiated earlier with respective decreases in λ values of 86 and 60% as they were faster with respective increments in *q* values of 8.8 and 10% (Table 2).

Regarding nitrite, during cycles 16–45, its accumulation was permanent up to 10 h of culture, reaching a final maximum concentration of 27.0 mg NO_2_^−^-N/L (Fig. 3b). This was accompanied by a decrease in the nitrate production in the same period (Fig. 3c). Final YNO_2_^−^ and YNO_3_^−^ values were 0.24 ± 0.01 and 0.75 ± 0.02 mg N/mg NH_4_^+^-N consumed, respectively (Table 1). At the same time, the qNO_3_^−^ decreased by 33% (Table 2). These results evidenced that although the ammonium oxidizing process was not altered by AMP addition, the nitrite-oxidizing process was clearly inhibited. Subsequently, 30 cycles (15 days) after the addition of AMP (cycles 46–75), the final NO_2_^−^-N accumulation disappeared, again presenting a transient accumulation profile (Fig. 3b; Table 1). NO_3_^−^-N was the main product of ammonium oxidation, reaching again YNO_3_^−^ values close to one (Fig. 3c; Table 1). During this same period, the inhibitory effect of AMP on nitrate formation disappeared, as the specific rates were like those obtained in the control period without antibiotic addition (Table 2). These results indicate that NOB were sensitive to AMP, but they were able to metabolically adapt to the toxic and/or inhibitory effect of the antibiotic after 30 cycles of operation. In the last evaluation period with AMP (cycles 76–87), as observed for the ammonium oxidation process, the nitrite oxidation process improved throughout the operation cycles (Fig. 3). The λ for nitrate formation decreased by 61% during cycles 76–87 compared to the control value (cycles 1–15) while there was an increment of 5% in qNO_3_^−^ between the same periods (Table 2). Initially, the negative effect of AMP on the activity of NOB was evident; however, as the cycles passed, they not only recovered their initial physiological activity, but they were also capable to increase their kinetic activity. In the study of Esquivel-Mackenzie et al. [28], the physiological adaptation of NOB to AMP inhibition in a SBR fed with 14.4 mg AMP/L took 30 days (30 cycles) and was associated with the increase in *Nitrospira* abundance.

## Conclusions

The present study provides new information on AMP removal by biotransformation and biosorption processes in a nitrifying reactor and on the role of nitrifying enzymes (AMO, NOR) and β-lactamase resistance enzymes in AMP biodegradation. Ampicillin at 50 mg/L was totally removed by the nitrifying sludge from the first operation cycle of the SBR reactor. The kinetic activity of the sludge for AMP removal improved throughout the cycles, reaching a complete removal after only 2 h. This improvement was associated with a higher ammonium-oxidizing capacity throughout the cycles. Biodegradation was the main process for AMP removal where the AMO enzyme contributed 95% to the cometabolic biotransformation. The ammonium oxidation process was not affected by the antibiotic addition, while the nitrite oxidation process was inhibited, causing nitrite accumulation in the SBR reactor. However, 30 operation cycles later, the NOB were able to totally recover their physiological and kinetic activity for oxidizing nitrite into nitrate. The nitrifying sludge did not acquire bacterial resistance by β-lactamase activity even after 72 cycles of operation under AMP exposure. These results indicate the role of AOB from nitrifying sludge in the biodegradation of AMP that can be used for bioremediation of contaminated water bodies.

## Data Availability

Data supporting the findings of this study are available within the paper. Should any raw data files be needed in another format they are available from the corresponding author upon reasonable request.
